# Parasites and politics: why cross-cultural studies must control for relatedness, proximity and covariation

**DOI:** 10.1098/rsos.181100

**Published:** 2018-08-29

**Authors:** Lindell Bromham, Xia Hua, Marcel Cardillo, Hilde Schneemann, Simon J. Greenhill

**Affiliations:** 1 Macroevolution and Macroecology, Division of Ecology and Evolution, Research School of Biology, Australian National University, Canberra, Australian Capital Territory 0200, Australia; 2 ARC Centre of Excellence for the Dynamics of Language, Australian National University, Canberra, Australian Capital Territory 0200, Australia; 3 Erasmus Mundus Master in Evolutionary Biology (MEME), Place E. Bataillon, Montpellier 34095, France; 4 Department of Linguistic and Cultural Evolution, Max Planck Institute for the Science of Human History, Kahlaische Strasse 10, Jena 07743, Germany

**Keywords:** Galton's problem, cultural evolution, phylogenetic comparative method, spatial autocorrelation, behavioural immune system

## Abstract

A growing number of studies seek to identify predictors of broad-scale patterns in human cultural diversity, but three sources of non-independence in human cultural variables can bias the results of cross-cultural studies. First, related cultures tend to have many traits in common, regardless of whether those traits are functionally linked. Second, societies in geographical proximity will share many aspects of culture, environment and demography. Third, many cultural traits covary, leading to spurious relationships between traits. Here, we demonstrate tractable methods for dealing with all three sources of bias. We use cross-cultural analyses of proposed associations between human cultural traits and parasite load to illustrate the potential problems of failing to correct for these three forms of statistical non-independence. Associations between parasite stress and sociosexuality, authoritarianism, democracy and language diversity are weak or absent once relatedness and proximity are taken into account, and parasite load has no more power to explain variation in traditionalism, religiosity and collectivism than other measures of biodiversity, climate or population size do. Without correction for statistical non-independence and covariation in cross-cultural analyses, we risk misinterpreting associations between culture and environment.

## 1. Introduction

The search for meaningful predictors of variation in human cultural traits and the diversity of human societies has a long history [1,2]. Modern analyses of broad-scale cultural diversity use an array of statistical analyses to extract patterns from global data, such as the effect of primary productivity on hunter–gatherer populations [3], the interaction between agricultural practises and religious beliefs [4] and the influence of rivers on language diversity [5]. However, many such analyses fail to account for one or more sources of statistical non-independence inherent in large observational datasets, which can lead to spurious relationships between traits and environments. Non-independence violates fundamental statistical assumptions and can lead to inflated degrees of freedom, incorrect parameter estimates, and the false impression of direct, causal relationships between variables that are only indirectly or incidentally linked. We can consider the problems of statistical non-independence in cross-cultural studies in three broad categories: phylogenetic non-independence, spatial autocorrelation and covariation among variables.

### 1.1. Galton's problem or non-independence due to evolutionary relationships

Human populations are related by descent, so closely related societies share many cultural traits that they have inherited from their shared ancestors. Societies that share a more recent common ancestor are likely to be more similar in many aspects of culture, including religious beliefs, material culture and social norms, than they are to more distantly related societies [6–8].

The statistical challenges of inferring cause–effect associations from comparisons of evolutionary outcomes have long been recognized. Darwin recognized that comparing species could lead to falsely interpreting correlation between traits which were incidentally inherited together from a common ancestor [9], a problem now referred to as phylogenetic non-independence or ‘Galton's problem’. In Galton's words, if cultural traits are derived from a common source, then they are duplicate copies of the original [2], rather than being independent outcomes of a causal mechanism. Galton's problem has also been expressed as the challenge of distinguishing ‘historical’ from ‘functional’ associations in cross-cultural analysis [10].

Most researchers in cultural evolution are aware of Galton's problem [11–14], but there has been a dearth of effective solutions. One proposed solution is the systematic selection of a set of cultures designed to limit the autocorrelation between samples [15]. For example, the Standard Cross-Cultural Sample represents a defined set of non-neighbouring cultures with rigorous sampling of cultural traits [16]. A sampling strategy such as this may reduce the amount of autocorrelation between cultural samples, but it does not solve the problem of phylogenetic non-independence, because cultures within the sample will still tend to be more similar to closer relatives in the database than more distant relatives [17]. For example, the cultures within the ‘Insular Pacific’ section of the Standard Cross-Cultural Sample database (such as Fijian, Maori and Trobriand Islanders) are likely to be more similar to each other in very many factors (culture, language, resources and climate) than any of them are to cultures in the ‘Circum-Mediterranean’ section (such as Turks, Armenians and Russians). The inclusion of a higher taxonomic level as a random grouping factor in mixed-model analyses provides a partial account of hierarchical structuring of data due to descent [13,18], but it fails to fully account for patterns of relatedness within, or between, the taxonomic groups [19]. Within any given section, sampled cultures will still cluster by relationship. For example, within the Insular Pacific section, we expect Maori and Marquesan to be more similar in most respects to each other than either is to the Balinese sample. Indeed, analyses suggest that around half of the variables in the Standard Cross-Cultural Sample show evidence of phylogenetic and spatial auto-correlation [17,20].

There are formal methods for accounting for patterns of relatedness in statistical analyses, and these are routinely used in evolutionary biology [19,21–27]. Given information about the relatedness between observations, statistically independent comparisons can be made between observations from species or cultures [28]. An explanatory model can be fitted to trait data by incorporating a covariance matrix that describes the non-independence of residuals that results from shared ancestry among the units of observation [29]. But most of these methods require a phylogeny that describes the evolutionary history of observations.

One of the factors that has inhibited the uptake of methods in the field of cultural evolution is that comprehensive phylogenies are not available for all human groups. There are few robust cultural phylogenies, and in some cases it may be difficult to express complex, intertwined cultural histories as a bifurcating phylogeny [30,31]. Unfortunately, lack of a phylogeny does not make lack of phylogenetic correction acceptable [28]. The *p*-values and parameter estimates from statistical tests are contingent on the assumption of independence of datapoints, and are therefore misleading when applied to traits related by descent without appropriately modelling covariation due to relatedness.

The capacity for human cultures to change rapidly and adapt to their current circumstances, thus potentially overcoming the constraints of their own history, is not in itself an argument against the need to correct for cultural history [32]. Instead, this argument implies a need to test whether traits of interest show clustering by relationships [33]. If related cultures show similar values, such that the residuals of groups of datapoints are correlated with each other, then standard methods that assume independence of residuals cannot be used, and phylogenetic correction must be applied. Unwillingness to assume a particular phylogenetic structure in a dataset is not a conservative approach: in fact, this is equivalent to making the radical assumption that the data are completely unstructured by historical patterns of descent. For example, failure to incorporate covariation due to relatedness assumes that the value of a cultural trait measured for The Netherlands is no more likely to be similar to the value of that trait for Belgium than to its value for Zimbabwe. Because cultural data often show strong hierarchical structuring [20], lack of correction for relatedness imposes a ludicrously unrealistic assumption on the analysis for most cultural traits.

Fortunately, there are approaches to correcting for relatedness between cultures that do not require a complete phylogeny, as long as some estimate of relatedness is available [34]. For example, comparisons between pairs of cultures can be considered phylogenetically independent if we can be certain that the members of each pair are more closely related to each other than they are to any other culture included in the study [35–37]. In this paper, we demonstrate an alternative approach, which is to construct a hierarchy of relationships based on readily available information and use that to inform estimates of covariation between cultures. We emphasize that, in the absence of a perfect fully resolved phylogeny, inclusion of any prior information on relatedness between cultures is an improvement on assuming that all cultures are equally closely related [38].

### 1.2. Spatial autocorrelation or non-independence due to proximity

In addition to considering the relatedness of cultures, we also need to take their spatial proximity into account, particularly when we are interested in the influence of environmental variables like climate, or aspects of biodiversity such as pathogen prevalence [3,39,40]. Neighbouring states are interconnected in many ways that can influence their cultural and social attributes, and shape the trajectories of cultural and economic change [41–43]. Neighbouring cultures will also share many aspects of environment with each other, including factors influencing subsistence (such as growing season) and human movement (such as river density) [5,44]. For example, cultural variables associated with subsistence are highly spatially autocorrelated in the Standard Cross-Cultural Sample dataset, as are measures of parasite load [17]. The spatial covariation between climate, environment, biodiversity and cultural traits creates the possibility of indirect statistical associations, whether or not there is a functional connection between them.

Accounting for spatial autocorrelation is sometimes approached by analysing data within regions, or by grouping observations into geographical regions or global ‘bands’ [5,10,45]. But, just as for phylogenetic non-independence, this grouping approach cannot fully remove spatial non-independence, because cultural samples within a region can also show significant spatial structuring which may cause spurious correlations between cultural, environmental and biodiversity variables [39,46,47].

Pseudoreplication arising from spatial autocorrelation is routinely addressed in macroecological studies, using a range of spatial analytical approaches [48]. These analytical tools have also been applied to the analysis of cultural data [3,46]. While these corrections are typically made on map-grid data, here we demonstrate how correction for spatial autocorrelation can use available information on the distance between sampled cultures. This approach can easily be applied to cross-cultural datasets where cultures are identified to geographically defined areas (e.g. countries) or to point-based localities.

### 1.3. Covariation or non-independence due to shared patterns among variables

As well as the particular problems of phylogenetic and spatial non-independence, many correlations involving cultural traits are confounded by covariation between traits of interest [49]. Covariation can cause variables to be significantly correlated with each other even if there is no direct causal connection between them. For example, any variable that has a latitudinal gradient will correlate with any other trait that shows a latitudinal gradient, even if the two variables are not causally connected. In some cases, bizarre combinations of variables can be correlated via indirect associations with other variables, such as a significant correlation between linguistic diversity and the annual number of road fatalities [7], or between chocolate consumption, Nobel prizes and IKEA stores [50,51]. In these cases, chains of intermediate linking factors can be found, such as gross domestic product, that generate indirect but statistically significant correlations between cultural and environmental traits [7,51].

The larger the dataset, the greater the capacity for spurious correlations [52], so a statistical test of association applied to a large dataset will not necessarily produce valid evidence with which to test a particular hypothesis, regardless of data quantity and quality. Covariation between variables is widely acknowledged, yet most broad-scale studies of cultural evolution include only a small selection of related variables. In this paper, we demonstrate the importance of simultaneous analysis of a wide range of relevant explanatory variables, including those representing cultural and environmental factors. In this way, the relative explanatory power of different hypotheses can be formally compared.

### 1.4. Case study: parasites and human cultural evolution

Analyses of the link between parasite prevalence and human cultural traits provide an important illustration of the combined influence of the three statistical problems described above. The influence of parasites on human cultural evolution has been viewed as an extension of a body of theory in evolutionary biology that describes the influence of parasite pressure on patterns of genetic variation and evolution in species [53,54]. For example, it has been suggested that parasite stress promotes the maintenance of genetic variation within populations, and drives sexual selection of indicators of good health and low parasite load [55,56], or causes rapid evolution through evolutionary arms races leading to population subdivision and divergence [57]. Parasite prevalence has also been suggested to influence human culture by favouring traits that promote in-group focus and exclusion of outgroup individuals [32]. The rationale behind this claim is that human groups may become relatively immune to local diseases, but remain vulnerable to parasites from other localities, so that limiting interaction with outsiders might reduce exposure to harmful pathogens [32]. Promotion of in-group cultural norms may serve to create barriers between groups to limit parasite exposure, including philopatry (lack of dispersal), xenophobia (dislike of out-group members), cultural conformity (enforcement of norms) and ethnocentrism (promoting interactions within the in-group) [32,58]. These traits are then proposed to have knock-on effects on a wide range of cultural traits, including both personal behaviour (such as openness to experience) and society-wide conventions (such as modes of governance).

The influence of parasite load on cultural evolution has been widely debated, and published studies have been criticized on methodological, empirical and practical grounds [43,49,59,60]. Our purpose here is to consider one specific criticism, which is that cross-cultural correlations showing a link between parasite load and cultural traits may be statistical artefacts [49]. Parasite stress has a distinct spatial pattern: there are more pathogen-caused human diseases in the tropics, and the potential for new zoonotic diseases increases with the diversity of their vertebrate hosts, which is also higher in the tropics [61]. In fact, the latitudinal diversity gradient in parasite stress is so pronounced that latitude has been used as a proxy for parasite load in some studies of cultural differences [45].

Cultural traits also have distinct non-random patterns as neighbouring cultures are likely to share more aspects of their history and environment with each other than with more distant cultures [7,62,63]. For example, language diversity shows a pronounced latitudinal gradient, and this is often interpreted in terms of the effect of climate on the size and stability of human cultural groups [44,47,64,65]. Any degree of spatial or genealogical clustering in the values of any particular cultural trait suggests a potential problem of statistical non-independence.

We demonstrate the pervasive influence of phylogenetic non-independence, spatial autocorrelation and covariation in the search for connections between parasite stress and cultural evolution using cross-cultural correlations. Our aim is not to provide a detailed critique of the hypothesis that parasite stress has been an important driver of human cultural evolution, so we will not examine the plausibility of the causal mechanisms suggested to underlie the observed patterns. We focus entirely on the statistical validity of one of the forms of evidence given in support of this hypothesis: cross-cultural comparisons.

## 2. Methods

Our aim is to ask whether the reported relationships between cultural variables and parasite load are robust to correction for the statistical non-independence of human cultural traits due to phylogenetic non-independence, spatial autocorrelation and covariation. Because we are reanalysing published data, we follow the convention of analysing at the level of nation states. We compiled four forms of data: (i) cultural variables reported to be associated with parasite load in previously published studies, for which data are available; (ii) measures of parasite load used in these studies; (iii) information on location, environmental variables and human population data for these states; and (iv) a representation of expected degree of relatedness between cultures.

**Figure 1.**
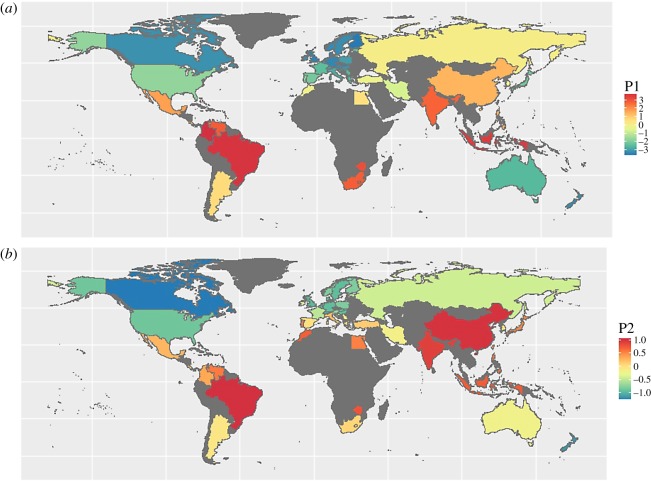
The 50 states included in this study, coloured by: (*a*) published values for Combined Parasite-Stress index (P1), on normalized scale of average disease prevalence; (*b*) values of Historical Pathogen Prevalence (P2), which is a combined index based on past epidemiological impact of nine human diseases (leishmaniasis, trypanosomes, malaria, schistosomes, filariae, leprosy, dengue, typhus and tuberculosis), on a normalized averaged scale.

Analysis at the state level introduces a set of possible biases—for example, the states vary greatly in geographical extent and population size, and the observations are not an evenly distributed sample of human cultures (figure 1). Because we reanalyse previously published datasets, our analysis reflects the geographical biases of previous studies, for example with relatively high sampling of European cultures but low sampling of African cultures [43]. But because state has been used in most cross-cultural studies of the influence of parasite load on human culture, it is the appropriate level for this reanalysis. We gathered available data for relevant cultural and environmental traits and then removed states for which 5 or fewer variables were recorded, leaving a final list of 50 states (electronic supplementary material, table S1). As our aim is to compare the relative explanatory power of different variables, our dataset balances number of states against number of variables. Some countries have missing data for some variables: these countries are not included in the analyses when these variables are used in the analyses.

### 2.1. Data

#### 2.1.1. Cultural variables

We used the published literature as a guide to selecting cultural traits that have previously been identified as correlates of parasite load, for which data were made available in the original publications (or their immediate sources: table 1). Where possible, we chose only one variable to represent each particular hypothesis. For example, Fincher *et al*. [70] include four different measures of ‘collectivism’ or ‘individualism’, derived from previous studies. Because these four variables are strongly correlated with each other [70], we selected the one with the greatest coverage in our dataset. We include four broad categories of cultural traits aligned by their explanatory roles.

(i) *Sexual behaviour:* Several studies have examined evidence that parasite prevalence might affect human mate choice, analogous to proposed effects on mate choice in animals [55,56]. For example, it has been suggested that high parasite load should lead to a greater importance placed on physical attractiveness as an indicator of health [55]. We have used data from Gangestad & Buss [55] who provide a composite measure of attractiveness for 37 countries, generated by asking individuals to rate the importance of various attributes as criteria for mate selection on a scale of 1 (low) to 3 (high). Both male and female averages were presented in that study, but here we use their ‘PA-TOT’ which is a sum of the two (labelled ATT in our analysis: table 1). Higher disease prevalence has also been suggested to lead to more restricted approach to sexual relationships in order to reduce exposure to pathogens [11]. We include the Sociosexual Orientation Inventory (SOI), a measure derived from self-reported attitudes to sexual activity and pair bonding. A high SOI score indicates an unrestricted, promiscuous attitude toward sexuality, a low score corresponds to a restricted, monogamous strategy. We use the data provided in Schmitt [77], labelled SEX in our analysis.
(ii) *Protective behaviour:* High parasite load has been suggested to drive behaviour that reduces pathogen exposure through adherence to group norms. For example, a recent study found that a measure of traditionalism correlated with historical pathogen prevalence, and they interpreted this as evidence of selection for rituals and rules that limit infection risk [67]. We include their published measures of traditionalism, based on surveys of individuals in 30 states (TRA: table 1). Similarly, obedience and conformity to accepted norms or behaviours that limit pathogen exposure is considered to be reflected in an association between parasite load and authoritarian forms of governance [68]. Two measures of authoritarianism are included in Murray *et al*. [68], but as both are highly correlated with each other, we include only the measure made from non-students (aut.nst, labelled AUT in table 1).
(iii) *Social structure*: High pathogen prevalence has been suggested to favour collectivist values, where loyalty to the group is valued over self-interest, in order to enforce behaviour that limits pathogen exposure [58,69]. We include two measures reflecting governance of group behaviour. Collectivism (COL) represents the degree to which groups are focused on in-group associations and emphasize conformity to group norms. As measures of individualism are highly negatively correlated with measures of collectivism, we selected the variable ‘Individual (Hofstede)’ [70] because it had the largest coverage for our dataset. As an alternative measure of social structure and governance, the Index of Democracy (DEM) reflects both competition and participation in electoral process [68].
(iv) *In-group bias:* Vulnerability to disease is considered to promote hostility towards outsiders (such as immigrants), in order to limit out-group contact and therefore exposure to novel pathogens [67,69]. Bias towards in-groups has been suggested to limit both gene flow and the flow of cultural information, thereby creating and maintaining linguistic and cultural diversity [57]. Of the four measures of in-group bias presented in Fincher & Thornhill [32], we use the measure of religiosity (REL) as the variable with the greatest coverage for our dataset [78]. Note that this is different from diversity of religions within a state. We include a measure of language diversity (LAN) for each state which is the count of languages in each country [79].

**Table 1.** Cultural traits that have been associated with parasite load, organized in broad categories, for which sufficient data were available to permit inclusion in this study. ‘Trait’ also includes the features of human populations and their environments that have been proposed as potential causes or correlates of variation in cultural traits. Label is the name given to each variable used in the analysis. Hypothesis gives a brief explanation of the proposed link and a reference where this hypothesis is discussed.

trait	variable	label	hypothesis	reference
sexual behaviour	attractiveness	ATT	high value placed on attractiveness as an indicator of health	[55]
	sociosexuality	SEX	disease risk favours restricted sexual contacts	[66]
protective behaviour	traditionalism	TRA	adherence to rules and rituals reduces pathogen risk	[67]
	authoritarianism	AUT	adherence to social structures and norms reduces exposure	[68]
in-group bias	language diversity	LAN	limited dispersal divides populations into small groups and reduces outgroup contact	[57]
	religiosity	REL	reduced association with people outside group limits exposure	[78]
social structure	collectivism	COL	threat from parasites favours obedience and social coordination	[69]
	democracy	DEM	institutionalized emphasis on conformity and ethnocentrism limits outgroup contact	[68]
pathogen stress	current	P1	current health burden and lifespan cost of infectious disease	[32]
	historical	P2	past epidemiological impact of key pathogens	[70]
population	population size	POP	group size can influence parasite transmission	[71]
	population density	DEN	population density can influence parasite diversity	[72]
environment	latitude	LAT	human pathogens increase in diversity with decreasing latitude	[73]
	temperature	TEM	temperature can influence pathogen prevalence and disease transmission	[74,75]
	growing season	GRO	reliable food production reduces group size and interaction between groups	[44]
biodiversity	bird diversity	BIR	diversity of hosts may increase diversity of parasites	[76]
	mammal diversity	MAM	pathogen prevalence is associated with bird and mammal species richness	[61]
	bird and mammal	BAM	combined species richness of birds and mammals	

#### 2.1.2. Parasite load

Because our aim is to examine the statistical robustness of previous results, we use previously published measures of contemporary or historical parasite load. Combined Parasite-Stress (P1: figure 1*a*) combines two sources of the burden of infectious disease from the World Health Organization (WHO): Infectious Disease Disability Adjusted Life Years, and a measure of morbidity and mortality attributed to 28 different infectious diseases [78]. Historical Pathogen Prevalence (P2: figure 1*b*) is based on past epidemiological impact of nine human diseases (leishmaniasis, trypanosomes, malaria, schistosomes, filariae, leprosy, dengue, typhus and tuberculosis) [70].

#### 2.1.3. Environmental and population variables

Parasite load has strong spatial patterning, to the extent that latitude has been used as a proxy for parasite load [45]. Latitude also correlates with some population and cultural variables [7,62–65]. As latitude predicts many environmental variables, such as growing season, precipitation and temperature seasonality, there is a potential for indirect relationships between climate, biodiversity, culture and latitude to generate significant correlations in cross-cultural datasets. Because of this, some studies of the effect of parasite stress on cultural variation control for latitude [55,66,69]. By adding in additional environmental variables, we can ask if any of them vary with culture above and beyond their covariation with latitude.

In order to control for covariation of cultural traits and parasite load with aspects of environment or human population, we derived a number of variables summarizing environmental conditions within each of the 50 states, including median latitude and mean annual temperature (table 1). The length of growing season has been suggested to influence human cultural evolution by determining group size and degree of contact between groups. Where growing seasons are long, lower ‘ecological risk’ means that small populations can be self-sufficient; smaller average population size promotes language diversity [44], which in turn has been linked to parasite diversity [57]. Pathogen prevalence may also be affected by population variables such as population size and density [80].

Variables for the 50 states were derived by using maps retrieved from the R package rworldmap [81,82]. Absolute latitude (LAT) was derived from the centroid of the state polygon using rworldmap. High-resolution data retrieved from BioClim were rescaled to a raster of grid cells of approximately 200 km × 200 km. The mean annual temperature of a country (TEM) was calculated as the average of the grid cells whose centre was within the state polygon [83]. We derived growing season (GRO) from GAEZ growing season index (http://www.fao.org/nr/gaez/en/). Growing season was not included for Egypt as the reported value (4 days) is an inappropriate reflection of an agricultural system built on irrigation from the Nile rather than rainfall (on which the measure of growing season is based).

Data on each state's human population size (POP) and population density (DEN) were derived from the World Bank (https://data.worldbank.org/indicator/, data downloaded 10 October 2017). We have used the 2016 data because historical figures before industrialization are not available for all states (the earliest World Bank records are from 1961). Taiwan is not in the World Bank list, so we have used the commonly reported figure of 649 people per square kilometre and 23.5 million people.

#### 2.1.4. Biodiversity

Parasite diversity, like many other aspects of biodiversity, tends to show predictable spatial patterns, especially with latitude [84,85]. Therefore, we expect parasite diversity to covary with many other measures of biodiversity and climate. We include species richness of both birds and mammals, as likely hosts for human pathogens, and the source of the majority of zoonotic diseases [80]. While the nature of the association between biodiversity and disease risk continues to be debated [86], increased zoonotic disease risk is associated with higher biodiversity, including mammal diversity [87]. More generally, biological, cultural and linguistic diversity have been suggested to show similar global patterns, potentially due to shared drivers of diversity [62].

To calculate bird and mammal species richness, we obtained species distribution maps from BirdLife International and NatureServe databases, downloaded via BiodiversityMapping.org. The maps were transformed to equal-area projections and overlaid onto a raster grid to calculate species richness within each grid cell. The summary measure of bird and mammal species richness for each country was the mean value of species richness across all of the 10 × 10 km grid cells whose centre lies within the boundary of the state polygon.

#### 2.1.5. Relatedness and proximity between cultures

To correct for spatial autocorrelation in cross-cultural analyses, we constructed a matrix of great circle distances between the centroids of all pairs of states. Even though there is currently no global phylogeny of cultures, we must correct for relatedness using whatever information is available to us to make a statement about expected patterns of relatedness between cultures (sometimes referred to as an ‘assumed phylogeny’). Languages provide a convenient proxy that tracks cultural evolution [8,17,38,41,88]. For example, linguistic relationships between states have significant explanatory signal for differences in governance style (on an autocracy/democracy spectrum) [41]. However, not all languages have available phylogenies, and the relationship between language families is a matter of debate [38,89,90]. Placing states in a hierarchy of relatedness using the taxonomy of each state's predominant language provides a convenient and tractable way to estimate covariance due to descent [38,40,41,88,91–93]. For each state, we identified the primary language spoken from information in Ethnologue [79]. Where there were multiple possible choices, we aimed to select the one that represented the largest majority of people in the country, and that provided the predominant cultural context. These choices are particularly difficult for states with a history of colonization, where there are many indigenous languages, but the language of the colonizers may have the largest number of speakers. In many cases, we have recorded the language of the colonizers because, even if it does not track the ethnic origins of all citizens of the state, it may reflect the dominant cultural and political context, which is relevant to many of the cultural variables in the dataset. No set of language choices will be perfect, but we aim for the best representation we can despite complex cultural histories (electronic supplementary material, table S2).

**Figure 2.**
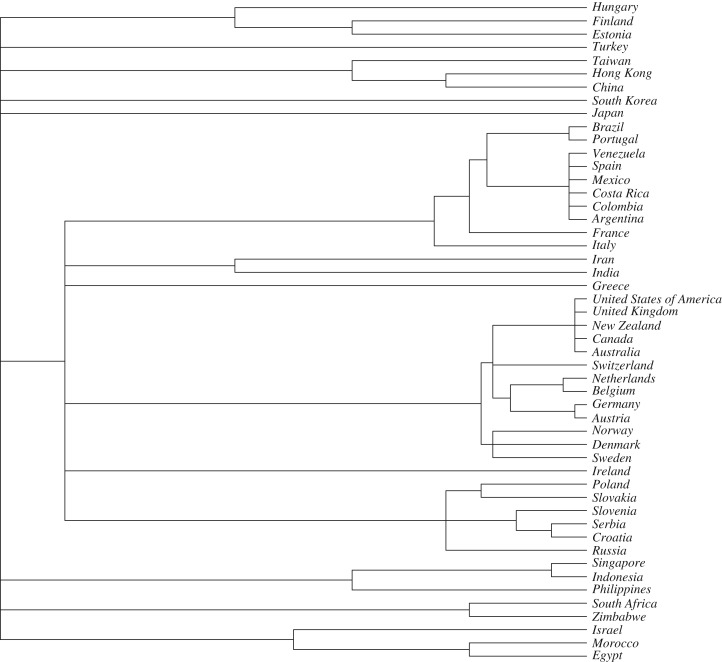
Cultural hierarchy used to estimate expected patterns of covariation due to relatedness, based on the taxonomy of the current dominant language. See §2.1.5 and electronic supplementary material (table S2) for details.

To arrange these languages in a hierarchy, we used the unique language identifier ISO-639-3 codes to place the languages in the taxonomy of all languages derived from Glottolog v3.0 [94], which provides an annotated classification of the world's languages based on expert assessment of their relationships, then represents this information as a ‘tree’. Because this tree is used to predict expected amount of cultural similarity, using unitary branch lengths that simply represent the number of nodes in the hierarchy will be misleading. It would suggest that all sister pairs of languages in the sample are expected to have the same degree of similarity to each other, which is clearly not the case for a small, non-random sample. For example, in our dataset, using unitary branch lengths would imply that we expect the same level of cultural similarity between Iran and India as between Netherlands and Belgium, or Canada and the United States (figure 2). To correct for this problem, we rescaled the height of nodes to provide a relative reflection of their dissimilarity, as follows. We identified the clades that included the sampled languages and recorded the number of languages in that clade from Glottolog (electronic supplementary material, table S2). We assigned a relative height to the ancestral node of each clade (*C*) that is proportional to the age of the language family from published phylogenies [95–101] and used this to scale the height of each clade relative to the root. Then for each clade, we counted the number of levels in the language classification between the root and the tips. We then used this to divide the clade height into arbitrary units representing depth of divergence within the clade. Then we scaled the node height within each language group by the classification level of tips, using the following formula:

$$(L_{c}-L_{s})\times C,$$

where *L*_c_ is the maximum classification level in the clade, *L*_s_ is the classification level containing the sampled languages and *C* is the clade height relative to the root.

### 2.2. Analysis

To summarize our approach, we first ask which of the cultural variables correlate with parasite load. Then we ask whether these correlations are robust to correction for phylogenetic and spatial non-independence. Once we have identified cultural variables that have a significant correlation with parasite load beyond that due to relatedness and proximity, we ask if these correlations can be explained by covariation between cultural variables. Then for any cultural variables that have a significant relationship with parasite load beyond that explained by proximity, relatedness and covariation, we examine whether those correlations are indicative of a causal relationship (variation in parasite load drives variation in cultural variables) or are suggestive of an indirect relationship (caused by covariation between both parasite load and cultural variables with other factors such as environment or population factors).

**Figure 3.**
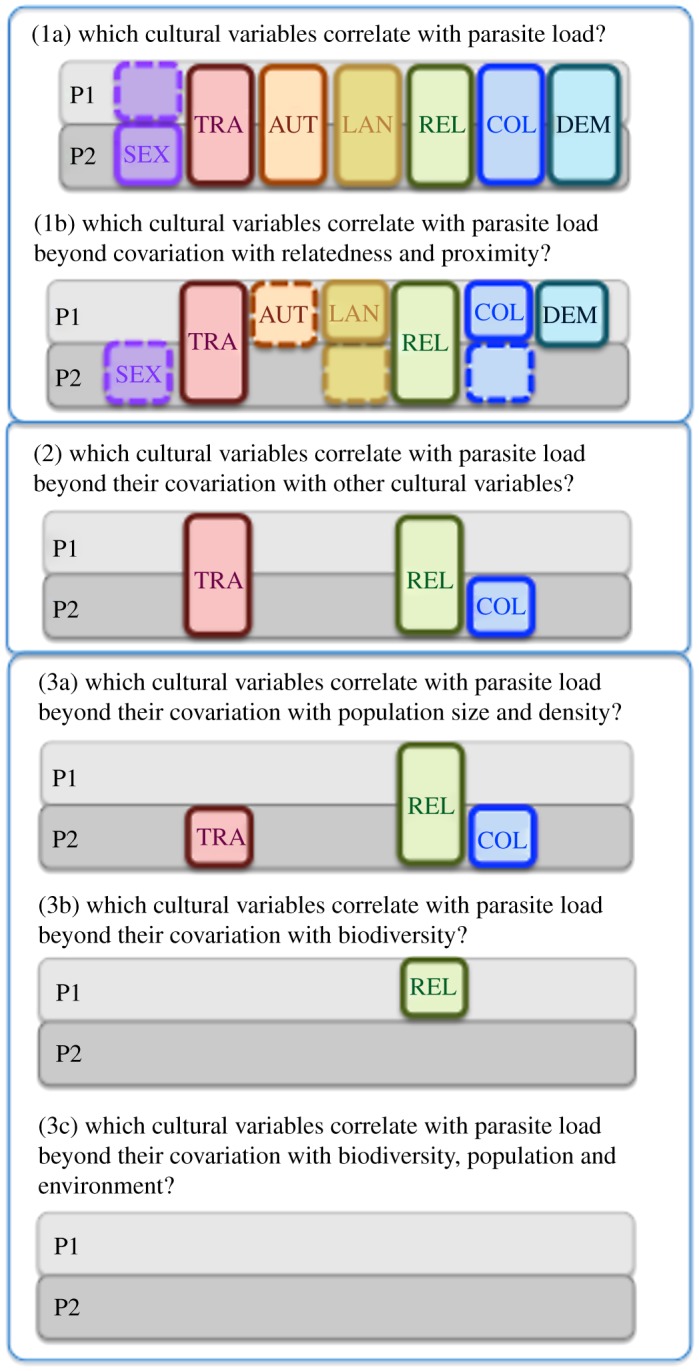
Schematic summary of the results of analyses. Boxes indicate a significant correlation between variables (table 1) and parasite load (*p* < 0.05). Dashed lines indicate results that would not be significant under a Bonferroni correction for multiple tests (*p* < 0.003). Details of statistical tests: (1, 2) table 3; (3) table 4; (3a) table 5; (3b) table 6; (3c) table 7.

To test for significant associations between variables, we fit generalized least-squares (GLS) models that simultaneously account for phylogenetic and spatial autocorrelation by incorporating both phylogenetic and spatial covariance matrices [102]. Likelihood ratio tests were performed to assess the relative fit of nested models and the Akaike information criterion (AIC) was used to assess the relative fit of non-nested models. We begin by analysing all variables, but any variable that is shown to have an incidental association with parasite load that arises from covariation between the variables and not due to a causal relationship is dropped from all further analysis (figure 3). This approach is similar to that advocated by Hrushka & Heinrich [103, p. 6], of ‘culling hypotheses through strategic model comparison rather than testing each hypothesis against a straw man null model’.

#### 2.2.1. Correcting for spatial autocorrelation and phylogenetic non-independence

We model the autocorrelation between data points (states) as a linear function of phylogenetic similarity and spatial similarity [102]. This is done by constraining the covariance between residuals of data points in GLS models to the linear function and estimating the contribution of phylogenetic similarity and spatial similarity simultaneously with the regression coefficients of the models. The overall contribution of phylogenetic and spatial similarities is estimated by one minus the ratio between the log-likelihoods of models that correct for phylogenetic and spatial similarities and models that do not correct phylogenetic and spatial similarities. Phylogenetic similarity is estimated by the correlation between states derived from the cultural hierarchy described above (figure 2). Spatial similarity is estimated by a Gaussian function over the great-circle distances between the centroids of all pairs of states, calculated using functions ‘gCentroid’ and ‘earth.dist’ from the R packages ‘rgeos’ and ‘fossil’, respectively [104,105]. In this way, the method simultaneously accounts for spatial and phylogenetic non-independence. Parameters in the autocorrelation function are estimated by maximizing the likelihood of the regression model using GLS implemented in R package ‘nlme’ [106]. We used ‘subplex’ method in the R package ‘nloptr’ [107] to find the maximum likelihood. Likelihood ratio tests were performed to assess the relative fit of nested regression models and AIC was used to assess the relative fit of non-nested regression models.

## 3. Results

### 3.1. Controlling for relatedness and proximity

First, we ask if each of the cultural variables correlates with either of the two alternative measures of parasite load (P1 and P2). When we conduct simple univariate GLS models, we find that all cultural traits correlate with both measures of parasite load, except for attractiveness (ATT: table 2, figure 3.1a). These results confirm that our dataset of 50 states is representative of the published literature because we get the same correlations between cultural traits and parasite load as previously reported (with the exception of attractiveness for which the original study had only 29 states included [55]).

**Table 2.** Pairwise association between cultural traits (the dependent variables, *y*) and two measures of parasite load (the independent variables, *x*). The regression coefficient (*β*), standard error, *t*-value, *p*-value and log-likelihood are given both for uncorrected regression (each datapoint is treated as a statistically independent observation) and corrected regression (correcting for the non-independence due to spatial autocorrelation and phylogenetic non-independence). The contribution of autocorrelation due to both phylogenetic and spatial similarities is calculated as 1−logLik(Corrected)/logLik(Uncorrected). Negative value suggests worse model fit when residual covariance between data points is constrained to a linear function between phylogenetic and spatial similarities. Results with *p* < 0.05 are given in bold. Results that would not be considered significant with Bonferroni adjusted *p*-values for 16 tests (*p* < 0.003) are given in italics.

			uncorrected					corrected					contribution of autocorrelation
*y*	*x*	d.f.	*β*	s.e.	*t*	*p*	logLik	*β*	s.e.	*t*	*p*	logLik	
ATT	P1	22	−0.022	0.039	−0.553	0.586	−17.734	−0.044	0.022	−2.040	0.053	−13.202	0.256
	P2	23	0.080	0.157	0.511	0.614	−18.134	0.342	0.211	1.620	0.119	−13.467	0.257
SEX	P1	29	−1.303	0.552	−2.363	***0***.***025***	−99.352	−1.203	0.597	−2.016	0.053	−97.408	0.020
	P2	31	−5.618	1.742	−3.225	**0**.**003**	−107.968	−4.528	2.192	−2.065	***0***.***047***	−104.532	0.032
TRA	P1	26	0.155	0.043	3.628	**0**.**001**	−15.602	0.147	0.044	3.326	**0**.**003**	−13.368	0.143
	P2	26	0.524	0.109	4.794	**0**.**000**	−12.470	0.528	0.113	4.690	**0**.**000**	−12.198	0.022
AUT	P1	27	0.390	0.095	4.094	**0**.**000**	−45.537	0.279	0.132	2.108	***0***.***044***	−55.147	−0.211
	P2	28	1.446	0.327	4.425	**0**.**000**	−46.469	0.734	0.475	1.546	0.133	−58.308	−0.255
LAN	P1	46	0.415	0.076	5.472	**0**.**000**	−78.279	0.382	0.087	4.371	**0**.**000**	−75.633	0.034
	P2	48	1.074	0.309	3.479	**0**.**001**	−88.226	0.774	0.326	2.374	***0***.***022***	−84.143	0.046
REL	P1	44	0.677	0.120	5.649	**0**.**000**	−95.300	0.544	0.117	4.664	**0**.**000**	−83.902	0.120
	P2	46	1.862	0.494	3.767	**0**.**001**	−106.495	2.104	0.023	91.129	**0**.**000**	−83.388	0.217
COL	P1	44	−6.151	1.163	−5.287	**0**.**000**	−198.540	−3.937	1.113	−3.537	**0**.**001**	−189.413	0.046
	P2	46	−24.776	3.847	−6.440	**0**.**000**	−204.192	−15.085	5.947	−2.536	***0***.***015***	−197.944	0.031
DEM	P1	27	−3.033	0.563	−5.387	**0**.**000**	−97.028	−2.241	0.599	−3.741	**0**.**001**	−93.487	0.036
	P2	27	−8.777	2.340	−3.752	**0**.**001**	−101.527	−0.605	3.072	−0.197	0.845	−97.344	0.041

When we take relatedness and spatial proximity into account (figure 3.1b), the relationship between parasite load (P1 and P2) and sociosexuality (SEX), authoritarianism (AUT), attractiveness (ATT) are either non-significant or marginally significant (table 2). However, language diversity (LAN), degree of democracy (DEM), traditionalism (TRA), collectivism (COL) and religiosity (REL) have significant relationships with parasite load (P1 and/or P2) that cannot be explained by spatial autocorrelation or phylogenetic non-independence. Given that there is no clear significant correlation between attractiveness and either measures of parasite load, ATT is dropped from any further analysis. Accounting for phylogenetic and spatial similarities increases model fit in all cultural variables except for AUT, explaining 2–26% of variation in the cultural variables (table 2).

### 3.2. Controlling for covariation between cultural variables

All of the remaining cultural variables covary with other cultural variables (table 3). This means that a relationship between parasite load and any one of these variables could generate significant associations between parasite load and some or all of the other variables, even in the absence of any direct causal connection. So we need to ask if the association between these traits and parasite load is simply due to covariation or whether there is evidence for independent association with parasite load above and beyond the relationships between the cultural variables. We fit a GLS model for each cultural trait, using the significantly covarying traits as independent variables, and use likelihood ratio tests to ask whether model fit improves with the addition of parasite load (P1 and P2: table 4). We only test combinations of variables that we have already identified as having significant correlations. For example, SEX is significantly associated with P2 (not P1: table 2), COL and DEM (table 3). But adding P2 to the model does not add significant explanatory power to the correlation between SEX and COL (table 4). We conclude that the association between SEX and parasite load can be explained by its covariation with COL, and that parasite load provides no additional explanation of variation in SEX (table 4).

**Table 3.** Pairwise correlation between cultural variables (see table 1 for variable names). Kendall's tau (*t*) for the correlation between each pair of cultural variables and the associated *p*-value are given. Significant *p*-values (*p* < 0.05) are indicated in bold.

	variable	SEX	TRA	AUT	LAN	REL	COL
TRA	*t*	−1.691					
	*p*	0.107					
AUT	*t*	−2.040	3.594				
	*p*	0.055	**0**.**002**				
LAN	*t*	−0.587	2.600	0.791			
	*p*	0.562	**0**.**015**	0.436			
REL	*t*	−0.325	0.931	2.658	2.827		
	*p*	0.747	0.361	**0**.**013**	**0**.**007**		
COL	*t*	2.476	−3.099	−4.094	−1.164	−1.763	
	*p*	**0**.**019**	**0**.**005**	**0.000**	0.251	0.085	
DEM	*t*	2.250	−3.024	−5.214	−0.689	−5.393	2.670
	*p*	**0**.**037**	**0**.**008**	**0**.**000**	0.497	**0**.**000**	**0**.**013**

**Table 4.** Does parasite load explain variation in cultural traits beyond covariation between cultural traits? For each cultural trait, we identify which other cultural traits it is significantly correlated with, then we fit a GLS model between those traits (Model 1). We use a likelihood ratio test to ask whether adding parasite load to the model (Model 2) improves the fit to the data. If the result is significant (in bold), then we conclude that parasite load explains variation in the cultural trait beyond its covariation with other cultural traits.

X	Model 1	lnL(M1)	Model 2	lnL(M2)	LR (M1/M2)
P1	AUT ∼ TRA	−28.355	AUT ∼ p1+TRA	−28.209	0.291
	LAN ∼ REL	−75.183	LAN ∼ p1+REL	−69.721	**10.924**
	LAN ∼ REL + TRA	−44.593	LAN ∼ p1 + REL + TRA	−42.610	3.967
	COL ∼ TRA	−111.831	COL ∼ p1+TRA	−110.610	2.441
	DEM ∼ TRA	−54.021	DEM ∼ p1+TRA	−54.015	0.012
P2	SEX ∼ COL	−101.543	SEX ∼ p2+COL	−100.649	1.788
	LAN ∼ REL	−82.084	LAN ∼ p2+REL	−80.390	3.387
	COL ∼ TRA	−111.831	COL ∼ p2+TRA	−108.468	**6**.**725**
	COL ∼ TRA + REL	−107.262	COL ∼ p2+TRA + REL	−103.938	**6**.**647**

For language diversity (LAN), adding P1 provides a better fit than the correlation between LAN and REL, but not when TRA was also added. Adding P2 did not provide a better fit to LAN than the correlation between LAN and REL, so we conclude that the relationship between parasite load and LAN is likely due to its covariation with TRA and REL, not a direct association between parasite load and language diversity (table 4).

For democracy (DEM) and authoritarianism (AUT), adding P1 to the model does not add any additional explanatory signal over their correlation with traditionalism (TRA: table 4). So we conclude that the association between DEM and AUT and parasite load is likely to be an indirect effect of the covariation between DEM, AUT and TRA (table 3), because TRA varies with parasite load (table 2).

For collectivism (COL), P1 does not give a significant increase in model fit over its covariation with traditionalism (TRA), but adding P2 does, and P2 also provides significantly better explanation of COL when both TRA and REL are added to the model (table 4). Therefore, we conclude that COL has a significant association with P2 above and beyond its covariation with TRA and REL. We do not test COL against REL because they are not significantly correlated with each other (table 3).

In summary, we find that the correlations between parasite load and language diversity, authoritarianism, sociosexuality and democracy can be accounted for by their covariation with religiosity, traditionalism and collectivism, which all have significant variation with parasite load beyond their covariation with other cultural traits. We have no evidence that parasite load has any explanatory power for variation in LAN, AUT, SEX and DEM, so we drop these variables from further analysis (figure 3.2).

### 3.3. Controlling for covariation with other factors

We have three variables remaining whose association with parasite load cannot be accounted for by phylogeny, spatial distribution or covariation between cultural traits: religiosity (REL), collectivism (COL) and traditionalism (TRA) (figure 3.2). Now, we wish to investigate the nature of the relationship between these cultural traits and parasite load: are the correlations indicative of a causal connection, or do they arise because both parasite load and cultural traits vary with some other factor, causing an indirect association between culture and parasites? We test a number of candidate variables that could correlate with both parasite load and cultural traits: population size and density, biodiversity and environmental variables.

#### 3.3.1. Population size and density

Parasite load is correlated with human population variables. Population size of nation states (POP) is significantly associated with both P1 and P2 (P1: *t* = 4.731, *p* < 0.001; P2: *t* = 3.718, *p* = 0.001). Population density (DEN) is significantly correlated with P2 (*t* = 2.052, *p* = 0.046), but not with P1 (*t* = −0.676, *p* = 0.502) or population size (*t* = −0.013, *p* = 0.990). We find that P2 has a significant association with COL, TRA and REL above and beyond its covariation with population size and density, and P1 has significant association with REL above and beyond its covariation with population size (table 5). So we conclude that the correlation between parasite load and cultural traits cannot be explained as an indirect effect of population parameters of the nation states (figure 3.3a).

**Table 5.** Does population size or density explain the relationship between cultural variables and parasite load? For each cultural trait (TRA, REL, COL) that has a significant association with parasite load (P1 and P2), a GLS model (Model 1) is fitted by using POP and/or DEN as the independent variables. A likelihood ratio test is then conducted to ask whether model fit to the data improves with the addition of parasite load (Model 2 for P1 and P2). If the result is significant (in bold), then we conclude that parasite load explains variation in the cultural trait beyond its covariation with population size and/or density.

X	Model 1	logL (M1)	Model 2	logL (M2)	LR(M1/M2)
P1	TRA ∼ POP	−16.509	TRA ∼ p1 ∼ POP	−14.778	3.462
	REL ∼ POP	−92.117	REL ∼ p1 ∼ POP	−83.109	**18.016**
P2	TRA ∼ POP + DEN	−15.175	TRA ∼ p2 ∼ POP + DEN	−10.891	**8**.**569**
	COL ∼ POP + DEN	−198.083	COL ∼ p2 ∼ POP + DEN	−192.679	**10**.**808**
	REL ∼ POP + DEN	−93.093	REL ∼ p2 ∼ POP + DEN	−84.772	**16**.**641**

#### 3.3.2. Biodiversity

Parasite load, like other measures of biodiversity, tends to show strong spatial patterns. For example, parasite load correlates with latitude (P1: *t* = −5.450, *p* < 0.001; P2: *t* = −2.260, *p* = 0.029), and so does biodiversity (BAM: *t* =−5.672, *p* < 0.001). Cultural traits are also correlated with latitude even after accounting for spatial proximity and relatedness among states (TRA: *t* = −3.692, *p* = 0.001, REL: *t* = −70.806, *p* < 0.001, COL: *t* = 3.972, *p* < 0.001). It is possible that there are direct links between vertebrate diversity and parasite diversity: more vertebrate species represent more hosts, which could result in more parasites [108]. If there is any association between biodiversity and cultural traits, then we would also expect it to generate an indirect association between parasite load and cultural traits. So we need to ask if parasite load provides a better explanation of cultural traits than other measures of biodiversity do.

Both measures of parasite load are significantly correlated with bird species richness (BIR: P1, *t* = 8.055, *p* < 0.001, P2: *t* = 5.47, *p* < 0.001) and mammal species richness (MAM: P1, *t* = 4.219, *p* < 0.001, P2: *t* = 2.771, *p* = 0.008), so we use the summation of bird and mammal species richness (BAM) as a measure of biodiversity (BAM: P1, *t* = 7.524, *p* < 0.001, P2: *t* = 3.908, *p* < 0.001).

Bird and mammal diversity (BAM) provides a better predictor of variation in traditionalism (TRA) than parasite load does. Using BAM as the only independent variable in a model of TRA gives better fit than using parasite load as the only independent variable (P1: ΔAIC = 14.460, P2: ΔAIC = 12.121, electronic supplementary material, table S8). We also find that parasite load does not provide a better explanation of variation in collectivism (COL) than other measures of biodiversity. Adding parasite load (P1 or P2) as an additional independent variable in a GLS model of COL or TRA against BAM does not improve the model fit, suggesting that the parasite load does not provide a better explanation of variation in COL and TRA than other measures of biodiversity (table 6). BAM has similar explanatory power for variation in COL as parasite load (P2: ΔAIC = 0.096, table 7). We conclude that the variation between states in two cultural traits—traditionalism and collectivism—can be equally well or better explained by the diversity of mammals and birds than by parasite load (figure 3.3b).

**Table 6.** Does biodiversity explain the relationship between cultural variables and parasite load? For each cultural trait (TRA, REL, COL) that has significant association with parasite load (P1 and P2), a GLS model (Model 1) is fitted by using a biodiversity measure BAM as the independent variable. A likelihood ratio test is then conducted to ask whether model fit to the data improves with the addition of parasite load (Model 2 for P1 and P2). If the result is significant (in bold), then we conclude that parasite load explains variation in the cultural trait beyond its covariation with an alternative measure of biodiversity. If the result is significant, we add other possible factors that covary with both cultural traits and parasite load to Model 1 (POP and GRO) and a likelihood ratio test is conducted to test if biodiversity together with these covarying factors can explain the association between cultural traits and parasite load.

X	Model 1	lnL(M1)	Model 2	lnL(M2)	LR (M1/M2)
P1	TRA ∼ BAM	−12.073	TRA ∼ p1+BAM	−12.070	0.006
	REL ∼ BAM	−86.816	REL ∼ p1+BAM	−83.834	**5.965**
	REL ∼ BAM+POP+GRO	−75.380	REL ∼ p1+BAM+POP+GRO	−73.707	3.345
P2	TRA ∼ BAM	−12.073	TRA ∼ p2+BAM	−12.060	0.026
	REL ∼ BAM	−90.469	REL ∼ P2+BAM	−89.318	2.302
	COL ∼ BAM	−197.992	COL ∼ P2+BAM	−196.094	3.796

**Table 7.** Do population, environment or biodiversity explain the relationship between cultural variables and parasite load? Comparisons of fit of models where cultural traits are linked to biodiversity (represented by BAM), environment (represented by LAT) and population (POP and DEN) against models that include parasite load (P1 and P2), as assessed with the AICs (ΔAIC_P1_, ΔAIC_P2_). A model with ΔAIC > 2 is considered to fit significantly better than a direct-link model with parasite load, and is highlighted in bold. If the ΔAIC_P1_ of one model is greater than ΔAIC_P1_ of another model by more than 2, we conclude it is a significantly better fit to the data (ditto comparing ΔAIC_P2_ of different models).

variable	model	ΔAIC_P1_	ΔAIC_P2_
biodiversity	TRA ∼ BAM	**14.460**	**12**.**121**
	REL ∼ BAM	−5.479	−18.118
	COL ∼ BAM	−1.475	−0.096
environment	TRA ∼ LAT	1.525	−0.813
	REL ∼ LAT	**13**.**675**	**3**.**226**
	COL ∼ LAT	1.688	**3**.**602**
population + environment	TRA ∼ POP + GRO	−4.135	−6.473
	REL ∼ POP + GRO	−17.825	−29.777
	COL ∼ POP + GRO	−3.571	−2.198
	TRA ∼ LAT + POP + GRO	1.435	−0.903
	REL ∼ LAT + POP + GRO	**13**.**216**	**6**.**499**
	COL ∼ LAT + POP + GRO	−0.033	1.890

However, adding P1 in a model of REL against biodiversity does improve the model fit (table 6). While religiosity also covaries with bird and mammal diversity, there is additional variation in REL that correlates with parasite load but cannot be explained by covariation with other biodiversity measures.

#### 3.3.3. Environmental variables

It is possible that the covariation between parasites, cultural traits and biodiversity is due to the independent influence of climate and other environmental variables. For example, parasite diversity, like other measures of biodiversity, is correlated with environmental factors such as temperature [109]. Human cultural diversity has also been suggested to be influenced by climatic factors: for example, areas with a long growing season and stable year round climate are predicted to favour division of human populations into smaller cultural groups with reduced spatial extent [44]. As biodiversity and climate tend to covary, we need to ask if the correlation between culture and biodiversity (including parasites) could be an indirect effect of covariation with environmental variables.

Growing season and temperature both vary with latitude (GRO: *t* = −3.198, *p* = 0.002, TEM: *t* = −17.017, *p* < 0.001), so first we ask if these environmental factors provide a better explanation of variation in parasite load than latitude does. GRO has additional explanatory power for P1 beyond its covariation with latitude (P1: *t* = −2.303, *p* = 0.026), but not P2 (P2: *t* = −1.770, *p* = 0.084). TEM has no additional explanatory power for variation in either P1 or P2 beyond its covariation with latitude (P1: *t* = −1.842, *p* = 0.072, P2: *t* = 1.229, *p* = 0.226, table 8).

**Table 8.** Do environment and population explain variation in parasite load beyond covariation with latitude? A GLS model (Model 1) is fitted to parasite load (P1 and P2) using latitude (LAT) and the environmental (TEM and GRO) or population variables (POP and DEN) that are significantly correlated with the parasite load measure. A likelihood ratio test is then conducted to ask whether removing these environmental or population variables significantly decreases the model fit to the parasite load (Model 2). If the result is significant, then we conclude that the environmental or population variables explain the variation in parasite load beyond the latitudinal gradient. When there are two environmental or population variables in the model, an additional likelihood ratio test is conducted to ask if removing one environmental or population variable significantly decreases the model fit. A significant result of the likelihood ratio test is highlighted in bold.

Model 1	logL (M1)	Model 2	logL (M2)	LR(M1/M2)
P1 ∼ LAT + TEM + GRO	−81.475	P1 ∼ LAT	−87.265	**11.580**
P1 ∼ LAT + TEM + GRO	−81.475	P1 ∼ LAT + TEM	−85.795	**8**.**640**
P1 ∼ LAT + TEM	−85.795	P1 ∼ LAT	−87.265	2.940
P2 ∼ LAT + TEM + GRO	−25.458	P2 ∼ LAT	−28.216	5.516
P1 ∼ LAT + POP	−79.888	P1 ∼ LAT	−87.265	**14**.**753**
P2 ∼ LAT + POP + DEN	−24.678	P2 ∼ LAT	−28.216	**7**.**077**
P2 ∼ LAT + POP + DEN	−24.678	P2 ∼ LAT + DEN	−28.132	**6**.**908**
P2 ∼ LAT + DEN	−28.132	P2 ∼ LAT	−28.216	0.169

Parasite load is related to host population size and density [110], and human population size and density also correlate with latitude (POP: *t* = −2.363, *p* = 0.022, DEN: *t* = −2.057, *p* = 0.045), so we need to investigate if population factors could provide a possible indirect link between parasite load and cultural traits. POP has additional explanatory power for P1 and P2 beyond its covariation with latitude (P1: *t* = 4.024, *p* < 0.001; P2: *t* = 2.611, *p* = 0.012, table 8). DEN has no additional explanatory power for variation in P2 beyond its covariation with latitude (*t* = 0.399, *p* = 0.692, table 8). So temperature and density are removed from our list of factors that could cause covariance between parasite load and cultural traits.

**Figure 4.**
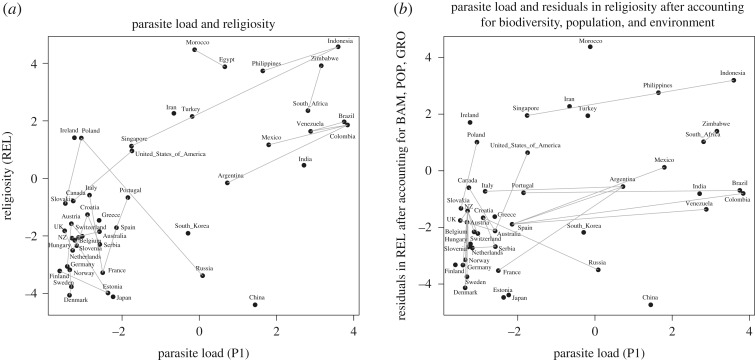
Example demonstrating phylogenetic and spatial non-independence in cultural data, with lines linking the states with the closest phylogenetic and spatial distance. For clarity, lines are only shown if autocorrelation is greater than 0.5. The strongest relationship between cultural traits and parasite load identified in this study is between religiosity and combined parasite stress (P1). (*a*) Autocorrelation patterns in the raw data. States with high autocorrelation in the lower left corner contribute to the positive correlation between parasite load and religiosity. After accounting for phylogenetic and spatial similarities between states, religiosity is still positively associated with parasite load across states. (*b*) After further accounting for covarying variables with both religiosity and parasite load (including biodiversity, population and environment), parasite load is not associated with the residuals in religiosity.

We now have two variables (population size and growing season) whose relationship with parasite load is not accounted for by other environmental factors, and we have an alternative biodiversity measure (BAM) that accounts for variation in cultural traits at least as well as parasite load, if not better. Given that biodiversity measures (bird, mammal and parasite richness) correlate with population size and growing season, then any cultural traits that also correlate with population size and growing season might show indirect correlations with parasite load. When we include BAM, POP and GRO in the model of REL, adding P1 does not provide significantly better explanation to REL, suggesting that parasite load and religiosity may be indirectly linked via population size, growing season and diversity of other species (table 6 and figure 4).

To distinguish indirect correlations from evidence for causal relationships, we need to ask which variables provide a better explanation of the variation in cultural traits, biodiversity or covarying factors, including latitude, population size and growing season. We find that bird and mammal species richness provides a significantly better explanation of variation in traditionalism than parasite load (P1 or P2), and latitude provides a significantly better explanation of the variation in religiosity and collectivism than biodiversity measures (bird, mammal and parasite species richness: table 7). Adding POP and GRO into the model does not significantly increase the explanatory power of variation in the three cultural traits (table 7).

#### 3.3.4. Summary

Our series of tests is aimed at comparing the relative explanatory power of parasite load, biodiversity, climate and population in explaining variation in human cultural traits. Because we are explicitly comparing published hypotheses, we are not seeking the best fit model from a multivariate analysis. Instead, our series of tests winnows the data, removing variables once we have demonstrated that their association with parasite load or cultural variation is better explained as the indirect result of covariation. The results of this series of tests suggest that while most of the cultural variables included in this study show an association with parasite load, these associations can all be accounted for by phylogenetic non-independence, spatial autocorrelation, covariation among cultural variables, and covariation with environment, biodiversity and population variables. The relationship between parasite load and culture is better explained by the covariation of traditionalism, religiosity and collectivism with biodiversity, population size and growing season, all of which correlate with both latitude and parasite load. We find no evidence to support the claim that parasite load provides a better explanation of human cultural variation than many other aspects of the environment.

## 4. Discussion

Parasite load correlates with aspects of human culture—but why? Our results suggest that we must be cautious in interpreting these cross-cultural correlations as a reflection of causal connections between parasite stress and the evolution of cultural traits. Previous tests of the relationship between parasite load and cultural traits have not corrected for the fact that closely related cultures, and those in geographical proximity, will share many aspects of culture, ecology and environment. These sources of non-independence in cross-cultural data can generate spurious or indirect correlations between cultural variables and parasite load. We find no evidence to support a significant role for parasite stress as a driver of cultural difference, because measures of parasite load provide no better explanation of cross-cultural variation than many other aspects of environment or biodiversity. As parasite load does not provide a better description of variation in cultural traits than many other environmental or biodiversity variables do, cross-cultural analyses provide no compelling reason to favour parasite stress as an explanation of cultural diversity.

Many cultural traits are intercorrelated, whether directly or indirectly: for example, religiosity correlates with number of languages per state, and degree of democracy correlates with sociosexuality (table 3). This means that a significant link between any one cultural trait and parasite load will tend to produce statistically significant correlations between parasite load and other cultural traits. Analysing variables separately, or in small groups of related variables, could lead to the impression that parasite load influences many different aspects of culture [59]. In particular, we find that language diversity, sociosexuality, democracy and authoritarianism have no significant correlation with parasite load, above and beyond that explained by their covariation with other cultural traits that scale with parasite load (traditionalism, religiosity, collectivism).

**Figure 5.**
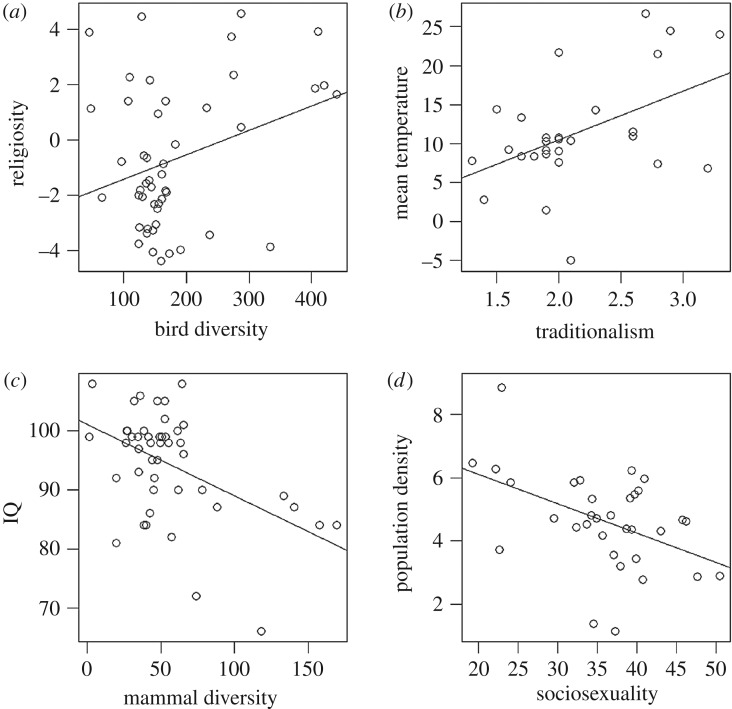
Examples of pairwise correlations between cultural and environmental variables. Data for up to 50 states from electronic supplementary material, table S1: (*a*) bird species richness versus religiosity (slope = 0.008, *t* = 2.12, *p* = 0.04); (*b*) mean yearly temperature versus traditionalism (slope = 6.26, *t* = 2.7, *p* = 0.012); (*c*) mammal diversity (species richness) versus average national IQ (slope = −0.12, *t* = −3.85, *p* < 0.001); (*d*) population density [log(DEN)] versus sociosexuality (slope = −0.09, *t* = −2.85, *p* = 0.008).

Covariation with shared environmental factors must also be accounted for. For example, because traditionalism shows a latitudinal gradient, it is likely to correlate with anything else that varies with latitude, such as mean temperature (figure 5*b*). A significant correlation does not tell us whether such correlations are indicative of causal relationships. For example, the significant association between sociosexuality and population density (figure 5*d*) could be interpreted as indicating a causal link (e.g. low availability of potential mates stimulates people to be more open to opportunities for sexual encounters) or an indirect link (e.g. both population density and sociosexuality decrease with latitude). Given the large number of factors that vary with latitude, there is little evidence that parasite load should be privileged as an explanation of cultural traits, especially as other factors (such as bird and mammal species richness, growing season and population size) provide as good or better explanation of variance in cultural traits in these data. The task of disentangling the associations between latitude and cultural traits is made more challenging by the relative underrepresentation of low-latitude countries in cross-cultural studies (figure 1).

It is important to note that there are two separate issues at hand here: whether two particular variables are correlated, and whether we should interpret a correlation as evidence for a causal association between the two variables. The correction for phylogenetic non-independence and spatial autocorrelation addresses the first of these issues, the tests of covariation address the second. There is no doubt that many of the cultural variables highlighted in the literature show a correlation with parasite load, but a statistically significant correlation and a plausible mechanistic link are not sufficient to establish causality. For example, the observed correlation between parasite load and IQ has been attributed to the metabolic costs of infection reducing investment in cognitive development [111]. IQ also correlates with environmental variables, such as temperature [111], and measures of biodiversity, such as mammal species richness (figure 5*c*), but we are not tempted to come up with a hypothesis to explain why having lots of mammal species reduces a nation's average IQ.

Correlations are effective tools for hypothesis generation, but are not always efficient tests of those hypotheses [7]. For example, it may be that parasite load does drive cultural evolution, but that other biodiversity and environmental variables are better predictors of parasite load than the variables used here (e.g. mammal and bird species richness may be more accurately known than parasite diversity). Nevertheless, our results show that, given the currently available data, we have no more reason to attribute the variation in cultural attributes to parasites than we do to latitude, environmental variation, population density or a number of other factors.

This paper is specifically a critique of the way in which cross-cultural correlation is frequently presented as evidence to support the hypothesis parasite load influences human cultural diversity, not a critique of the hypothesis itself. Other lines of evidence have been brought to bear on the ‘behavioural immune system’ hypothesis [112], such as ‘priming’ studies that test whether subjects' opinions or reactions are influenced by exposure to cues associated with pathogen exposure [113]. The validity or otherwise of those supporting behavioural studies is independent of the cross-cultural evidence. However, the existence of a plausible causal link, or independent experimental evidence, does not mitigate the problems of interpreting the cross-cultural evidence for the hypothesis. For example, the global correlation between bird diversity and religiosity (figure 5*a*) is supported by smaller scale studies showing that local bird diversity has a positive relationship with traditional culture in Tibet [114], and religiously significant sites have higher bird diversity in some areas [115,116]. However, the spatial distribution of major religions is non-random with respect to biodiversity hotspots [117], and religious diversity has been shown to correlate with other aspects of biodiversity, such as plant diversity [118], so we would be reluctant to accept a statistically significant correlation as convincing evidence that bird diversity drives religiosity, without teasing apart potential covarying factors and spatial autocorrelation.

Correcting for statistical biases is necessary to avoid being led astray by interpreting incidental associations as meaningful causal connections. It has been suggested that the behavioural immune system hypothesis may form a general law by which we might understand the course of human history, such as the occurrence of wars and authoritarian regimes, and the patterns of human diversity, such as religion and marriage practices, as well as the prevalence of individual traits including sexual behaviour, intelligence, open-mindedness and altruism, using only a few causal variables [119]. Given the wide reach and non-trivial implications of the observed correlations between parasite load and cultural traits, it is critical that the evidence used to support it is rigorously interrogated.

## Supplementary Material

Supplementary Material

## Data accessibility

All data analysed are available in the electronic supplementary material, and all data sources are cited.
