# Supplementary material for: Parasites and politics: why cross-cultural studies must control for relatedness, proximity and covariation
Source: R Soc Open Sci. 2018 Aug 29;5(8):181100. doi: 10.1098/rsos.181100 (PMC6124128; doi:10.1098/rsos.181100)
Supplement: Supplementary Material [file rsos181100supp1.docx]

Supplementary material for: **Parasites and politics: why cross-cultural studies must control for relatedness, proximity and covariation.**

Lindell Bromham, Xia Hua, Marcel Cardillo, Hilde Schneeman, Simon J. Greenhill

**Table S1: Data used in this study.** In addition to the variables listed in Table 1, the average IQ score (IQ) is used in supplementary analyses. Details of variables given in the Supplementary Methods. Not all variables were available for all 50 states.

| **State** | **P1** | **P2** | **ATT** | **SEX** | **TRA** | **AUT** | **LAN** | **REL** | **DEN** | **LAT** | **TEM** | **POP** | **COL** | **DEM** | **GRO** | **BIR** | **MAM** | **BAM** | **IQ** |
| --- | --- | --- | --- | --- | --- | --- | --- | --- | --- | --- | --- | --- | --- | --- | --- | --- | --- | --- | --- |
| Argentina | 0.73 | -0.07 |  | 40.7 | 1.5 |  | 17 | -0.16 | 16.02 | -35.20 | 14.42 | 43847430 | 46 |  | 151.7 | 181.5 | 35.1 | 216.7 | 93 |
| Australia | -2.59 | -0.20 |  | 37.3 | 2.0 | 4.34 | 146 | -1.85 | 3.14 | -25.58 | 21.66 | 24127159 | 90 | 37.1 | 79.1 | 166.2 | 26.6 | 192.8 | 98 |
| Austria | -3.31 | -0.72 |  | 45.7 |  | 4.49 | 5 | -1.58 | 106.00 | 47.59 | 6.45 | 8747358 | 55 | 37.7 | 202.6 | 135.6 | 61.5 | 197.1 | 100 |
| Belgium | -3.13 | -0.93 | 3.06 | 32.8 | 1.9 | 4.36 | 8 | -2.06 | 374.77 | 50.64 | 8.62 | 11348159 | 75 | 42.8 | 233.2 | 129.4 | 49.0 | 178.4 | 99 |
| Brazil | 3.75 | 1.02 | 3.57 | 37.9 | 2.9 | 4.47 | 167 | 1.97 | 24.84 | -10.77 | 24.50 | 207652865 | 38 | 24.5 | 290.8 | 419.6 | 140.5 | 560.1 | 87 |
| Canada | -3.26 | -1.29 |  | 34.5 | 2.1 | 4.31 | 78 | -0.78 | 3.99 | 57.78 | -4.98 | 36286425 | 80 | 24.9 | 106.8 | 95.9 | 30.6 | 126.5 | 99 |
| China | 1.45 | 1.00 | 3.65 |  | 3.2 |  | 209 | -4.39 | 146.85 | 36.62 | 6.75 | 1378665000 | 20 |  | 149.3 | 158.8 | 47.7 | 206.5 | 105 |
| Colombia | 3.84 | 0.35 | 2.78 |  |  |  | 75 | 1.86 | 43.85 | 3.91 | 24.70 | 48653419 | 13 |  | 335.4 | 405.5 | 157.7 | 563.2 | 84 |
| Costa Rica | 1.19 | 0.15 |  |  |  | 4.40 | 7 |  | 95.13 | 9.97 | 23.11 | 4857274 | 15 | 20.9 | 315.3 | 352.8 | 133.3 | 486.1 | 89 |
| Croatia | -2.90 | -0.34 |  | 43.0 | 2.0 |  | 1 | -1.26 | 74.53 | 45.13 | 10.57 | 4170600 | 33 |  | 231.9 | 160.7 | 61.8 | 222.6 | 90 |
| Denmark | -3.36 | -0.93 |  |  | 1.3 |  | 4 | -4.06 | 135.61 | 56.27 | 7.79 | 5731118 | 74 |  | 210.6 | 146.0 | 26.5 | 172.5 | 98 |
| Egypt | 0.66 | 0.54 |  |  |  | 4.65 | 8 | 3.88 | 96.13 | 26.56 | 22.06 | 95688681 |  | 3.0 |  | 44.2 | 19.6 | 63.7 | 81 |
| Estonia | -2.37 | -0.55 | 3.90 | 40.0 |  | 4.53 | 2 | -3.98 | 31.06 | 58.69 | 5.43 | 1316481 | 60 | 24.0 | 174.7 | 190.7 | 41.6 | 232.3 | 99 |
| Finland | -3.62 | -0.80 | 2.55 | 50.5 | 1.9 | 4.23 | 7 | -3.22 | 18.08 | 64.52 | 1.42 | 5495096 | 63 | 33.6 | 144.4 | 138.4 | 34.5 | 172.8 | 99 |
| France | -2.50 | -0.51 | 3.84 | 36.7 | 1.9 | 4.37 | 12 | -3.28 | 122.17 | 46.63 | 10.76 | 66896109 | 71 | 27.7 | 250.0 | 146.0 | 55.1 | 201.0 | 98 |
| Germany | -3.42 | -0.93 | 3.24 | 39.7 | 1.7 | 4.26 | 7 | -3.06 | 236.94 | 51.09 | 8.39 | 82667685 | 67 | 35.5 | 216.2 | 150.8 | 50.7 | 201.5 | 99 |
| Greece | -2.59 | 0.15 | 4.16 | 32.4 | 2.3 | 4.48 | 10 | -1.46 | 83.37 | 39.48 | 14.32 | 10746740 | 35 | 36.1 | 201.1 | 139.6 | 45.4 | 185.1 | 92 |
| Hong Kong |  | 0.32 |  | 22.9 |  | 4.59 | 2 | -3.88 | 6996.86 | 22.42 | 22.47 | 7346700 | 25 |  | 288.0 | 333.6 | 64.4 | 398.0 | 108 |
| Hungary | -3.22 | -0.93 |  |  |  | 4.25 | 5 | -2.15 | 108.45 | 47.17 | 10.20 | 9817958 | 80 | 25.4 | 189.2 | 160.3 | 63.6 | 223.9 | 98 |
| India | 2.71 | 0.90 | 4.00 |  | 3.3 | 4.72 | 437 | 0.46 | 445.37 | 22.90 | 24.00 | 1324171354 | 48 | 17.1 | 162.3 | 287.4 | 57.1 | 344.5 | 82 |
| Indonesia | 3.60 | 0.72 | 3.17 |  |  |  | 687 | 4.57 | 144.14 | -0.19 | 25.07 | 261115456 | 14 |  | 352.0 | 286.9 | 88.0 | 374.9 | 87 |
| Iran | -0.66 | -0.17 | 3.76 |  |  |  | 39 | 2.26 | 49.29 | 32.57 | 16.97 | 80277428 | 41 |  | 63.4 | 108.9 | 40.2 | 149.0 | 84 |
| Ireland | -3.23 | -0.39 | 3.09 |  | 1.6 |  | 2 | 1.41 | 69.29 | 53.18 | 9.23 | 4773095 | 70 |  | 319.1 | 106.1 | 20.0 | 126.1 | 92 |
| Israel | -2.83 | 0.60 |  | 41.0 | 2.8 | 4.59 | 6 |  | 394.97 | 31.44 | 21.53 | 8547100 | 54 | 26.4 | 124.0 | 165.7 | 47.9 | 213.6 | 95 |
| Italy | -2.84 | 0.22 | 3.64 | 34.4 |  | 4.23 | 22 | -0.58 | 206.03 | 43.53 | 12.18 | 60600590 | 76 | 36.8 | 239.1 | 132.1 | 52.5 | 184.6 | 102 |
| Japan | -2.23 | 0.51 | 2.59 | 24.1 | 2.6 |  | 12 | -4.12 | 348.35 | 36.65 | 10.92 | 126994511 | 46 |  | 259.7 | 173.0 | 32.0 | 205.0 | 105 |
| Mexico | 1.80 | 0.26 |  | 35.7 |  | 4.57 | 277 | 1.17 | 65.61 | 23.93 | 20.36 | 127540423 | 30 | 21.2 | 130.4 | 232.1 | 77.9 | 310.0 | 90 |
| Morocco | -0.12 | 0.70 |  | 39.3 |  |  | 9 | 4.47 | 79.04 | 31.88 | 17.23 | 35276786 | 46 |  | 98.8 | 128.5 | 38.7 | 167.2 | 84 |
| Netherlands | -3.28 | -0.93 | 2.97 | 39.3 | 1.9 | 4.17 | 12 | -2.50 | 505.15 | 52.23 | 9.13 | 17018408 | 80 | 38.4 | 243.1 | 153.1 | 38.5 | 191.6 | 100 |
| New Zealand | -3.30 | -1.16 |  | 47.7 | 2.1 | 4.35 | 2 | -2.08 | 17.82 | -43.99 | 10.37 | 4692700 | 79 | 33.5 | 312.2 | 64.6 | 1.4 | 71.5 | 99 |
| Norway | -3.35 | -0.80 | 3.19 |  |  | 4.27 | 6 | -3.18 | 14.33 | 64.24 | 1.77 | 5232929 | 69 | 39.2 | 136.6 | 124.9 | 26.7 | 151.6 | 100 |
| Philippines | 1.64 | 0.59 |  | 32.1 |  |  | 172 | 3.74 | 346.51 | 15.95 | 25.75 | 103320222 | 32 |  | 335.8 | 271.8 | 42.8 | 314.6 | 86 |
| Poland | -3.06 | -0.80 | 3.70 | 34.2 | 2.0 | 4.58 | 3 | 1.40 | 123.94 | 52.12 | 7.62 | 37948016 | 60 | 20.8 | 212.3 | 166.2 | 53.1 | 219.4 | 99 |
| Portugal | -1.85 | 0.56 |  | 29.6 |  |  | 6 | -0.67 | 112.71 | 39.68 | 15.27 | 10324611 | 27 |  | 241.5 | 136.5 | 44.0 | 180.5 | 95 |
| Russia | 0.09 | -0.42 |  |  |  | 4.62 | 94 | -3.38 | 8.81 | 61.66 | -5.11 | 144342396 | 39 | 28.0 | 115.8 | 137.0 | 34.7 | 171.7 | 97 |
| Serbia | -2.58 | -0.14 | 3.94 | 38.7 | 1.9 | 4.58 | 8 | -2.29 | 80.69 | 44.03 | 10.22 | 7095383 | 25 | 22.8 | 183.8 | 155.2 | 61.6 | 216.8 |  |
| Singapore | -1.76 | 0.36 |  |  | 2.7 |  | 24 | 1.12 | 7908.72 | 1.36 | 26.70 | 5535002 | 20 |  | 366.0 | 46.4 | 3.8 | 50.2 | 108 |
| Slovakia | -3.47 | -0.93 |  | 34.9 | 2.8 |  | 6 | -0.87 | 112.90 | 48.71 | 7.43 | 5423801 | 52 |  | 191.7 | 162.7 | 65.4 | 228.1 | 96 |
| Slovenia | -3.17 | -0.80 |  | 46.3 |  |  | 2 | -2.34 | 102.52 | 46.11 | 8.15 | 2063531 | 27 |  | 204.4 | 149.1 | 70.1 | 219.2 |  |
| South Africa | 2.81 | 0.09 | 2.05 |  |  | 4.53 | 13 | 2.35 | 46.09 | -29.01 | 17.19 | 55011976 | 65 | 12.5 | 96.1 | 275.6 | 74.2 | 349.9 | 72 |
| South Korea | -0.29 | 0.00 |  | 22.2 | 2.6 |  | 1 | -1.91 | 525.70 | 36.45 | 11.51 | 51014947 | 18 |  | 250.4 | 168.1 | 35.8 | 203.9 | 106 |
| Spain | -2.15 | 0.03 | 3.15 | 33.7 | 1.7 | 4.36 | 9 | -1.71 | 92.85 | 40.39 | 13.33 | 46447697 | 51 | 31.7 | 200.5 | 143.5 | 43.2 | 186.7 | 98 |
| Sweden | -3.31 | -0.93 | 3.11 |  | 1.4 | 4.34 | 7 | -3.77 | 24.31 | 62.84 | 2.83 | 9799186 | 71 | 37.7 | 149.4 | 123.2 | 30.4 | 153.6 | 99 |
| Switzerland | -3.03 | -1.04 |  | 39.1 |  |  | 7 | -2.01 | 211.87 | 46.81 | 4.00 | 8282396 | 68 |  | 172.0 | 123.7 | 65.6 | 189.3 | 101 |
| Taiwan |  | 0.34 | 3.04 | 19.2 |  |  | 17 | -3.43 | 649.00 | 23.75 | 20.09 | 23464787 | 17 |  | 327.6 | 236.5 | 52.6 | 289.1 | 105 |
| Turkey | -0.18 | 0.15 |  |  | 2.0 |  | 16 | 2.15 | 103.31 | 38.99 | 10.77 | 78271472 | 37 |  | 134.9 | 141.4 | 45.4 | 186.8 | 90 |
| United Kingdom | -3.49 | -0.96 | 3.32 | 40.2 | 1.8 | 4.40 | 5 | -1.82 | 271.31 | 53.94 | 8.33 | 65128861 | 89 | 33.5 | 240.8 | 126.0 | 27.6 | 153.6 | 100 |
| United States of America | -1.74 | -0.86 |  | 37.1 | 2.0 | 4.33 | 134 | 0.96 | 35.32 | 39.50 | 9.04 | 320896618 | 91 | 34.4 | 150.3 | 153.9 | 49.5 | 203.4 | 98 |
| Venezuela | 2.88 | 0.57 | 3.03 |  |  |  | 36 | 1.64 | 35.79 | 7.12 | 25.55 | 31568179 | 12 |  | 280.5 | 440.6 | 169.4 | 610.0 | 84 |
| Zimbabwe | 3.16 | 0.77 |  | 22.7 |  | 4.66 | 16 | 3.92 | 41.75 | -19.00 | 20.68 | 16150362 |  | 4.3 | 125.8 | 410.2 | 118.0 | 528.1 | 66 |

**Table S2:** Languages chosen to place each state in a cultural similarity hierarchy. Clade depth is derived from the relative node heights in published phylogenies [1-7]. Clade depth is the basal node height expressed as an approximate proportion of the root to tip distance.

| **State** | **Representative language** | **Glottocode** | **iso** | **Language Family** | **Family size** | **Clade**  **(Glottolog)** | **Clade size** | **Clade depth** |
| --- | --- | --- | --- | --- | --- | --- | --- | --- |
| **Argentina** | Spanish | stan1288 | spa | Indo-European | 584 | Italic | 86 | 0.4 |
| **Australia** | English | stan1289 | eng | Indo-European | 584 | Germanic | 106 | 0.2 |
| **Austria** | German | stand1295 | deu | Indo-European | 584 | Germanic | 106 | 0.2 |
| **Belgium** | Vlaams | vlaa1240 | vls | Indo-European | 584 | Germanic | 106 | 0.2 |
| **Brazil** | Portuguese | port1283 | por | Indo-European | 584 | Italic | 86 | 0.4 |
| **Canada** | English | stan1290 | eng | Indo-European | 584 | Germanic | 106 | 0.2 |
| **China** | Mandarin | mand1415 | cmn | Sino-Tibetan | 486 | Sinitic | 26 | 0.4 |
| **Colombia** | Spanish | stan1288 | spa | Indo-European | 584 | Italic | 86 | 0.4 |
| **Costa Rica** | Spanish | stan1288 | spa | Indo-European | 584 | Italic | 86 | 0.4 |
| **Croatia** | Croatian | croa1245 | hrv | Indo-European | 584 | Balto-Slavic | 25 | 0.3 |
| **Denmark** | Danish | dani1285 | dan | Indo-European | 584 | Germanic | 106 | 0.2 |
| **Egypt** | Egyptian Arabic | egyp1253 | arz | Afro-Asiatic | 374 | Central Semitic | 74 | 0.5 |
| **Estonia** | Estonian | esto1258 | ekk | Uralic | 48 | Uralic | 48 | 0.6 |
| **Finland** | Finnish | finn1318 | fin | Uralic | 48 | Uralic | 48 | 0.6 |
| **France** | French | stan1290 | fra | Indo-European | 584 | Italic | 86 | 0.4 |
| **Germany** | German | stan1295 | deu | Indo-European | 584 | Germanic | 106 | 0.2 |
| **Greece** | Modern Greek | mode1248 | ell | Indo-European | 584 | Greek | 9 | 0.6 |
| **Hong Kong** | Cantonese | cant1236 | yue | Sino-Tibetan | 486 | Sinitic | 26 | 0.4 |
| **Hungary** | Hungarian | hung1274 | hun | Uralic | 48 | Uralic | 48 | 0.6 |
| **India** | Hindi | hind1269 | hin | Indo-European | 584 | Indo-Iranian | 318 | 0.6 |
| **Indonesia** | Indonesian | indo1316 | ind | Austronesian | 1274 | Malayo-Polynesian | 1254 | 0.4 |
| **Iran** | Persian | fars1254 | fas | Indo-European | 584 | Indo-Iranian | 318 | 0.6 |
| **Ireland** | Irish | iris1253 | gle | Indo-European | 584 | Celtic | 16 | 0.4 |
| **Israel** | Modern Hebrew | hebr1245 | heb | Afro-Asiatic | 374 | Central Semitic | 74 | 0.5 |
| **Italy** | Italian | ital1282 | ita | Indo-European | 584 | Italic | 86 | 0.4 |
| **Japan** | Japanese | nucl1643 | jpn | Japonic | 15 | Japonic | 15 | 0.3 |
| **Mexico** | Spanish | stan1288 | spa | Indo-European | 584 | Italic | 86 | 0.4 |
| **Morocco** | Moroccan Arabic | moro1292 | ary | Afro-Asiatic | 374 | Central Semitic | 74 | 0.5 |
| **Netherlands** | Dutch | dutc1256 | nld | Indo-European | 584 | Germanic | 106 | 0.2 |
| **New Zealand** | English | stan1291 | eng | Indo-European | 584 | Germanic | 106 | 0.2 |
| **Norway** | Norwegian | norw1258 | nor | Indo-European | 584 | Germanic | 106 | 0.2 |
| **Philippines** | Tagalog | fili1244 | fil | Austronesian | 1274 | Malayo-Polynesian | 1254 | 0.4 |
| **Poland** | Polish | poli1260 | pol | Indo-European | 584 | Balto-Slavic | 25 | 0.3 |
| **Portugal** | Portuguese | port1283 | por | Indo-European | 584 | Italic | 86 | 0.4 |
| **Russia** | Russian | russ1263 | rus | Indo-European | 584 | Balto-Slavic | 25 | 0.3 |
| **Serbia** | Serbian | serb1264 | srp | Indo-European | 584 | Balto-Slavic | 25 | 0.3 |
| **Singapore** | Malay | mala1479 | zsm | Austronesian | 1274 | Malayo-Polynesian | 1254 | 0.4 |
| **Slovakia** | Slovak | slov1269 | slk | Indo-European | 584 | Balto-Slavic | 25 | 0.3 |
| **Slovenia** | Slovenian | slov1268 | slv | Indo-European | 584 | Balto-Slavic | 25 | 0.3 |
| **South Africa** | Zulu | zulu1248 | zul | Atlantic-Congo | 1433 | East Bantu | 252 | 0.2 |
| **South Korea** | Korean | kore1280 | kor | Koreanic | 2 | - | - | - |
| **Spain** | Spanish | stan1288 | spa | Indo-European | 584 | Italic | 86 | 0.4 |
| **Sweden** | Swedish | swed1254 | swe | Indo-European | 584 | Germanic | 106 | 0.2 |
| **Switzerland** | Swiss German | swis1247 | gsw | Indo-European | 584 | Germanic | 106 | 0.2 |
| **Taiwan** | Min Nan Chinese | minn1241 | non | Sino-Tibetan | 486 | Sinitic | 26 | 0.4 |
| **Turkey** | Turkish | nucl1301 | tur | Turkic | 43 | - | - | - |
| **United Kingdom** | English | stan1292 | eng | Indo-European | 584 | Germanic | 106 | 0.2 |
| **United States of America** | English | stan1293 | eng | Indo-European | 584 | Germanic | 106 | 0.2 |
| **Venezuela** | Spanish | stan1288 | spa | Indo-European | 584 | Italic | 86 | 0.4 |
| **Zimbabwe** | Shona | shon1251 | sna | Atlantic-Congo | 1433 | East Bantu | 252 | 0.2 |

**Supplementary references**

1. Bouckaert R, Lemey P, Dunn M, Greenhill SJ, Alekseyenko AV, Drummond AJ, Gray RD, Suchard MA, Atkinson QD. 2012 Mapping the origins and expansion of the Indo-European language family. *Science*. **337**, 957-960.

2. Grollemund R, Branford S, Bostoen K, Meade A, Venditti C, Pagel M. 2015 Bantu expansion shows that habitat alters the route and pace of human dispersals. *Proceedings of the National Academy of Sciences*. **112**, 13296-13301. (10.1073/pnas.1503793112)

3. Kitchen A, Ehret C, Assefa S, Mulligan CJ. 2009 Bayesian phylogenetic analysis of Semitic languages identifies an Early Bronze Age origin of Semitic in the Near East. *Proceedings of the Royal Society B: Biological Sciences*. **276**, 2703-2710.

4. Gray RD, Drummond AJ, Greenhill SJ. 2009 Language phylogenies reveal expansion pulses and pauses in Pacific settlement. *Science*. **323**, 479-483. (10.1126/science.1166858)

5. Honkola T, Vesakoski O, Korhonen K, Lehtinen J, Syrjänen K, Wahlberg N. 2013 Cultural and climatic changes shape the evolutionary history of the Uralic languages. *Journal of evolutionary biology*. **26**, 1244-1253.

6. Blench R, Post MW. 2014 Re-thinking Sino-Tibetan phylogeny from the perspective of North East Indian languages. *Trans-Himalayan Linguistics*. **266**, 71-104.

7. Lee S, Hasegawa T. 2011 Bayesian phylogenetic analysis supports an agricultural origin of Japonic languages. *Proceedings of the Royal Society of London B: Biological Sciences*. rspb20110518.
